# Axillary Nodal Staging with Contrast-Enhanced Ultrasound

**DOI:** 10.1007/s12609-017-0258-3

**Published:** 2017-11-04

**Authors:** Nisha Sharma, Karina Cox

**Affiliations:** 1 Breast Unit, Level 1 Chancellor Wing, St James Hospital, Beckett Street, Leeds, LS9 7TF UK; 2 Department of Breast Surgery, Maidstone Hospital, Hermitage Lane, Maidstone, Kent ME16 9QQ UK; 3 University of Leeds, Leeds, LS2 9JT UK

**Keywords:** Contrast-enhanced ultrasound (CEUS), Axilla, Breast cancer, Sentinel lymph node biopsy, Axillary surgery, Axillary ultrasound

## Abstract

**Purpose of Review:**

Axillary staging in the context of breast cancer is a contentious topic due to the varied practices across UK, Europe, and America. The ACOSOG Z0011 trial has questioned the role of axillary ultrasound in women with breast cancer. Published data has shown that women with ultrasound-positive lymph nodes have a worse prognosis than those with ultrasound-negative lymph nodes. Axillary ultrasound is limited as the sentinel lymph node (SLN) cannot be identified using B-mode ultrasound; however, with the advent of contrast-enhanced ultrasound (CEUS), this has now changed.

**Recent Findings:**

The published literature has shown that the sentinel lymph node can be identified using CEUS. The rates are equivalent to blue dye alone but currently inferior to the dual technique of sentinel lymph node biopsy. There are several different contrast agents that can be used and the agents that remain in the sentinel lymph node for longer can identify areas of poor enhancement, allowing for targeted biopsy.

**Summary:**

CEUS has the potential to revolutionize the way we manage the axilla in the future and may even replace surgical staging.

## Introduction

In patients with breast cancer, the identification of malignant axillary lymph nodes (LN) remains an important prognostic factor, which provides valuable information about overall survival to help guide adjuvant treatment decisions [1]. In many countries, axillary gray-scale ultrasound is a key component of the diagnostic pathway, but there are marked differences across the world in terms of which patients are offered routine axillary imaging.

In the UK and Europe, all newly diagnosed patients will have an axillary ultrasound to stage the axilla, even when clinical examination of the axilla is normal. Axillary lymph nodes that are equivocal, suspicious, or abnormal are biopsied. The biopsy can be performed by either conventional core biopsy or fine needle aspiration (FNAC). If the biopsy is benign, the patient is managed with a surgical sentinel lymph node biopsy (SLNB), and if malignant, an axillary lymph node dissection (ALND) [2–4]. In the USA, the guidelines recommend that an axillary ultrasound should only be performed in those patients with clinically palpable axillary lymph node [5], despite the fact that clinical palpation has a false-negative rate of 30–50% [6, 7]. These inter-continental differences are not surprising given that conventional B-mode axillary ultrasound has limited sensitivity and specificity with two recent meta-analyses suggesting that routine preoperative axillary ultrasound combined with core biopsy or FNAC correctly identifies nodal metastases in 50–55% of breast cancer patients [8, 9]. One in four patients with a negative axillary ultrasound, with or without a biopsy, is proven to have metastatic lymph nodes on subsequent SLNB. In view of this, SLNB is currently considered the gold standard for axillary staging.

SLNB has a high detection rate of 95% when the dual localization methods of blue dye and radioisotope are used [10]. However, the dual tracer SLNB technique may not be widely available around the world due to the high costs and logistical challenges of obtaining medical grade radioisotopes. Many centers use blue dye as the sole tracer but it is inferior to the dual technique [10] and has known problems such as a 0.9% risk of anaphylaxis [11]. The SLNB is also a surgical procedure, and although it has less morbidity than ALND, it is associated with debilitating complications such as sensory loss (11%) and arm lymphedema (5%) at 12 months [12]. There is now a strong imperative to find alternative methods to achieve reliable axillary staging that are less invasive and not dependent upon radioisotopes.

## Contrast-Enhanced Ultrasound and Identification of Sentinel Lymph Nodes in Patients with Breast Cancer

Contrast-enhanced ultrasound is well established in abdominal imaging [13] but contrast-enhanced ultrasound (CEUS) of the axilla is a relatively new technique that was first introduced in humans by Omoto et al. [14] to identify sentinel lymph nodes (SLN) under ultrasound guidance using 25% albumin solution as the contrast agent. In brief, the current procedure is performed under standard sterile technique with approximately 2 ml of 1% lidocaine injected subcutaneously in the upper outer quadrant of the breast, adjacent to the nipple. Then, an intradermal injection of 1 ml of the ultrasound contrast agent was performed using a 1-ml tuberculin syringe, raising a small bleb in the skin. The breast is gently massaged to encourage uptake of the microbubbles into the lymphatic system. Using contrast pulse sequencing, the breast ultrasound is performed starting at the periareolar region looking for the microbubbles in the lymphatic channel. Once the microbubbles are seen in the lymphatics, then this is followed in real-time to the axilla. The first axillary lymph node that fills with the ultrasound contrast is assumed to be the sentinel lymph node. A biopsy of this lymph node is performed and a clip placement is inserted in the lymph node.

This technology has progressed with the development of second-generation ultrasound contrast agents such as SonoVue (Bracco, Milano, Italy), Sonazoid (GE Healthcare, Oslo, Norway) and Definity (marketed in North America as Luminity by Lantheus Medical Imaging, North Billerica, MA, USA). These newer products stabilize the microbubbles by using an inert gas rather than air, which increases the transit time. This allows for real-time high-resolution imaging of both the arterial and parenchymal phases of enhancement within the lymph nodes. These contrast agents allow visualization of the lymph node microvessels as opposed to just the macrovessels with Doppler ultrasound. The two commonest agents used are SonoVue and Sonazoid. SonoVue consists of sulfur hexafluoride within a phospholipid shell. This is an inert molecule that does not interact with any other molecule in the body. After destruction of the microbubble, the gas is excreted through the lungs without any excretion through the kidney or the liver. Sonazoid consists of perfluorobutane within a hydrogenated egg phophatidylserine shell.

The two contrast agents differ in that the transit time from the time of injection to the time the agents reach the SLN is about 15–45 s with SonoVue and on average of about 5 min with Sonazoid [33]. The Sonazoid stays within the lymph node much longer, allowing for real-time localization of the SLN under ultrasound guidance, whereas the SonoVue contrast agent remains in the lymph node for 1–3 min. Shimazu et al. (2016) [15] compared CEUS using Sonazoid with SLNB using blue dye and/or radioisotope. The identification rate of SLN was 98% with CEUS and 100% with standard technique. They also noted that Sonazoid and CEUS identified significantly lower numbers of SLN compared with the tracers used for the surgical procedure. They postulated that because the mean diameter of the Sonazoid microbubble ranged from 2.4 to 3.5 μm, which is larger than that of the blue dye or radioisotope, the microbubbles do not readily traffic into the lymphatic system. Once trapped in the SLN, the microbubbles are retained and are unlikely to travel to lymph nodes further up to the lymphatic chain. A recent systematic review and meta-analysis of CEUS-guided SLN identification in breast cancer patients confirm that the technique is reproducible [16]. This review consisted of 11 prospective and one retrospective study with 1520 patients included from 2006 to 2015 with different methods used to validate the technique against SLN identification at the time of surgery. The various techniques used for preoperative localization of SLN included identification of the needle biopsy tract and/or marker clip placement at the time of histological assessment, radioactive iodine seed placement, and wire localization. The SLN identification and localization rate for CEUS-guided skin marking ranged from 70 to 100%, CEUS-guided-wire localization ranged from 89 to 97%, and CEUS-guided iodine-125 (I-125) seed localization was 60%. Across the four studies that evaluated preoperative CEUS-guided SLN biopsy, the pooled sensitivity for the identification of nodal metastases was 54% (95% confidence interval [CI] 47**–**61) and the pooled specificity was 100% (95% CI 99**–**100). In swine models, lymph node metastases can be seen as areas devoid of enhancement [17]. In a recent clinical study, Fei et al. (2015) [18] further refined the technique by looking at enhancement patterns within SLN and classifying them: type 1 where the SLN obviously enhanced and there was homogeneous enhancement; type 2 where the SLN obviously enhanced but the enhancement was not homogeneous, with hypoperfusion or non-perfusion areas; and type 3 where the SLN weakly enhanced or did not enhance. They found that type 1 was most commonly associated with non-malignant sentinel nodes and type 2 enhancement was more common in malignant sentinel lymph nodes. If they classified the enhancement patterns such that type 1 indicates negative nodes and type 2 and 3 indicate malignant nodes, then they achieved a sensitivity of 81.8%, specificity 86.2%, and accuracy rate of 84.7%. The positive predictive value was 75% and negative predictive value was 90.3%. Using CEUS to remove the SLN with a percutaneous device is feasible but perhaps surprisingly, does not appreciably increase the sensitivity of the technique as a test to identify SLN metastases. It can also negatively affect subsequent surgical operations in the axilla [19].

### Axillary Conservation and Sentinel Lymph Node Identification with CEUS

Following the publication of the ACOSOG Z0011 trial [20], there has been a renewed focus on axillary overtreatment. The trial showed that ALND can be safely omitted in selected patients with malignant SLN excised at SLNB. Multiple studies have shown that 40–70% of SLN-positive patients do not have further lymph node metastases found at the time of completion of ALND [12, 21, 22]. These studies all recruited patients with clinically normal axillary lymph node or a normal axillary gray-scale ultrasound. In a recent study by Verheuvel et al. [23], a difference in survival was noted between women with ultrasound-detected malignant lymph node and those with malignant lymph node detected after the surgical procedure (SLNB). The study compared patient, tumor, and lymph node characteristics and the results showed that patients with a malignant lymph node detected on ultrasound were more likely to have clinically palpable lymphadenopathy and larger tumors with worse prognostic factors, such as a higher tumor grade and lymphovascular invasion. Similar differences were observed after the exclusion of those with clinically palpable axillary nodes. In 2016, Cox et al. published a study in which 52% of patients with a normal gray-scale axillary ultrasound and a malignant SLN identified with CEUS had high volume axillary disease (two or more axillary lymph node macrometastases) identified at the end of primary surgical treatment [24]. This is an important finding which adds weight to the argument that lymph node found to be sonographically malignant tends to have a higher nodal burden at time of surgery [25, 26]. The Cox et al. study involved a large consecutive series of patients with the SLN identified in 93% of cases. All patients had a core biopsy of the SLN prior to surgery and lymph node tissue was retrieved in 555 patients. The prevalence of axillary lymph node metastases in the 555 patients was 23% (16.8% macrometastases, 4.5% micrometastases, and 1.8% isolated tumor cells). Of these, CEUS identified 53% (confidence interval (CI) 44–62%) of SLN metastases with 100% (CI 99–100%) specificity. The negative predictive value was 88%, and given a benign SLN biopsy result, the post-test probability that a patient had SLN metastases at subsequent surgical excision was 12%. They also found that only 2% of patients with a benign SLN core biopsy using CEUS had high volume axillary metastases identified at the end of primary surgical treatment [24].

### Current Challenges with CEUS Identification of SLN

There are two main challenges with using CEUS to target SLN in breast cancer patients. The first issue is that of reliable and consistent SLN identification as there can be intra-operative variability even when the same ultrasound equipment is used. Advances such as super-resolution imaging [27], ultrafast ultrasound [28], and improved microbubble transit [29] may improve the visualization of lymphatics and SLN.

Core biopsy is the preferred method to sample the SLN [8] but it can be technically challenging. Often, the nodes identified are small and not previously demonstrated on conventional ultrasound [30]. Injecting local anesthesia into the SLN allows the node to swell, which makes visualization easier for the core biopsy and clip placement if required (personal communication, Dr. Sharma 2017).

Choy et al. [31] showed that sterile black carbon suspension (Spot™) could be used to tattoo the lymph node sampled under ultrasound guidance. In their study, 16 women with suspicious axillary lymph nodes underwent ultrasound-guided core biopsy or FNAC, followed by injection of 0.1 to 0.5 ml of Spot™ ink into the cortex of the lymph node sampled. They concluded that the tattooed nodes were visible intra-operatively. This is an exciting development because it can be incorporated into a pathway for CEUS-guided SLN biopsy. The operating surgeon will be able to tell in real-time if the node sampled is within the SLNB or axillary clearance specimen and this would help support the accuracy of CEUS in SLN identification.

## Future Developments

Enhanced radiological staging of the axilla using CEUS in breast cancer patients is a reproducible technique that yields important information which can aid clinical decision-making. The sensitivity of the technique as a test to identify SLN metastases is close to 50% but the negative predictive value is high, which means that patients with a normal gray-scale ultrasound and benign CEUS-guided SLN core biopsy are unlikely to have ultrasound occult metastatic disease in the axilla. In the era of axillary conservation, the practice of surgically removing all malignant axillary lymph node may be an outdated concept as modern adjuvant therapies also target metastatic axillary disease. As the available evidence indicates that very few patients with a normal gray-scale ultrasound and benign CEUS-guided SLN core biopsy have high volume axillary metastases, these patients may be able to completely avoid axillary surgery. Conversely, the identification of patients with high volume axillary metastases early on in the diagnostic pathway may prove beneficial in terms of initiating systemic therapy. Complete axillary treatment with ALND and radiotherapy may also be appropriate to achieve local control [32].

## Conclusion

The technology of lymphatic mapping using CEUS can only improve. Superior ultrasound resolution and better contrast agents will allow consistent visualization of enhancement patterns in the SLN to facilitate targeted biopsy of localized metastases to improve the false-negative rate. A technique that could easily be adopted worldwide and is much cheaper than a surgical procedure would ensure standardized practice both in developed and developing countries. A large multi-center trial should be performed to validate the role of CEUS-guided SLN biopsy and ensure that it is not inferior to SLNB with regard to important oncological outcomes.
